# Nested-channel for on-demand alternation between electrospray ionization regimes†

**DOI:** 10.1039/d0sc06221a

**Published:** 2020-12-22

**Authors:** Mengtian Li, Huishan Li, Nicholas R. Allen, Taoqing Wang, Linfan Li, Jae Schwartz, Anyin Li

**Affiliations:** Department of Chemistry, University of New Hampshire 23 Academic Way Durham NH 03824 USA Anyin.Li@unh.edu; Thermo Fisher Scientific 355 River Oaks Pkwy San Jose CA 95134 USA

## Abstract

On-demand electrospray ionization from different liquid channels in the same emitter was realized using filamented capillary and gas phase charge supply. The solution sub-channel was formed when back-filling solution to the emitter tip by capillary action along the filament. Gas phase charge carriers were used to trigger electrospray ionization from the solution meniscus at the tip. The meniscus at the tip opening may be fully filled or partially empty to generate electrospray ionization in main-channel regime and sub-channel regime, respectively. For emitters with 4 μm tip opening, the two nested electrospray (nested-ESI) channels accommodated ESI flow rates ranging from 50 pL min^−1^ to 150 nL min^−1^. The platform enabled on-demand regime alternations within one sample run, in which the sub-channel regime generated smaller charged droplets. Ionization efficiencies for saccharides, glycopeptide, and proteins were enhanced in the sub-channel regime. Non-specific salt adducts were reduced and identified by regime alternation. Surprisingly, the sub-channel regime produced more uniform responses for a peptide mixture whose relative ionization efficiencies were insensitive to ESI conditions in previous picoelectrospray study. The nested channels also allowed effective washing of emitter tip for multiple sampling and analysis operations.

## Abstract 2

Nested electrospray ionization alternates on-demand between microscale main-channel and nanscale sub-channels.

## Graphical abstract

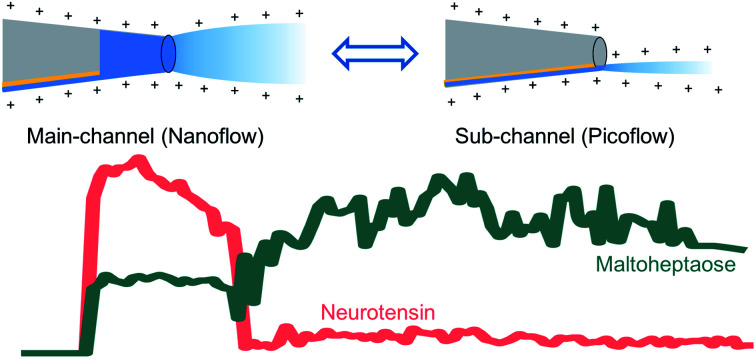

## Introduction

Electrospray ionization (ESI) is a soft ionization method that produces intact molecular ions from solution phase samples.^1^ It is extensively applied in mass spectrometry (MS) analysis of organic and biological samples. An existing challenge of ESI is that ionization efficiency of analyte is flow-dependent and sample-dependent.^2^ While accurate control and prediction of the ionization efficiency has not been achieved, ESI at lower flow rates has been found to provide higher analytical sensitivity, reduce ionization suppression and improve sample utilization efficiency.^3,4^ One critical aspect of lower flow rate is that it produces initial ESI droplets of smaller sizes that bear more charge per unit volume. Smaller charged droplets also require fewer fission events prior to the generation of gas phase ions.^5^ These provide sensitivity enhancement for analytes that are disfavored in charge competition during the evolution of ESI.^6^ Developments in the past three decades have brought methods to perform ESI at microflow, nanoflow, and more recently at picoflow.^7–9^ The sensitivity enhancement at lower flow rate has been demonstrated for saccharides,^10^ glycopeptides and glycoproteins,^11^ and antibodies.^12^ Beyond higher ionization efficiency, smaller charged droplets also play significant roles in eliminating non-specific adducts,^13,14^ and in the study of accelerated reaction kinetics.^15^

Pulsed DC and inductive AC electrospray has been demonstrated improved sample utilization efficiency with reduced flow rate to 30–160 pL min^−1^.^16,17^ However, pulsed ion sources are complicated by the possibility that the flow rates, droplet sizes and ionization efficiencies may vary at different time points in each pulse. For continuous ESI methods, a lower flow rate relies on the use of smaller emitter tips.^18^ 20–40 nL min^−1^ was considered to be the lower limit of flow rate range for continuous self-fed ESI using heat-pulled micrometer-sized emitter tips.^8,9^ However, flow rates of submicron emitter tips and nanofluidic slot (50 × 300 nm) achieved 0.75 nL min^−1^,^19^ and 0.11 nL min^−1^ respectively.^20^ External pump-driven ESI could reduce flow rates for micrometer-sized emitter tip down to 0.3 nL min^−1^.^9,21^ Although a wealth of methods have been developed in the electrochemical field to manufacture nanopipette,^22^ the efforts of lowering the ESI flow rate by using smaller emitter tips have been constrained by practical obstacles such as emitter clogging,^23^ nanometer tip fabrication,^24^ and sample handling.^13^

Besides using the circular opening of the capillary tips to form ESI meniscus that covers the whole tip opening, other emitter configurations have been found to be effective as well to forming the ESI meniscus. Ambient ionization methods^25^ have successfully demonstrated electrospray from the meniscus at the edge of flat surfaces.^26,27^ If such surface-supported meniscus can be formed inside an emitter tip, it will be much smaller than the tip opening and ideal for generating ESI with lower flow rates and smaller droplets.

Here we report a system and methods to generate ESI from meniscus smaller than the size of the emitter tip. The emitter was partially filled to perform continuous ESI at flow rates down to 50 pL min^−1^, defined as the sub-channel regime. When fully filled, the same emitter was demonstrated to also support nanoflow ESI up to 150 nL min^−1^, defined as the main-channel regime. The system allows rapid alternation between sub-channel and main-channel regimes in the same experiment, which reliably demonstrated the reduced initial droplet size and improved ionization efficiency in the sub-channel regimes. The presence of two separate channel also enabled the more complicated washing operations.

## Results and discussion

Sample solution was delivered to an emitter tip by capillary action along an inner-filament (160 μm O.D. glass rod) annealed to the inner wall of a glass capillary (0.89 mm I.D.). In the taper region, while the glass capillary was heat-pulled to form an emitter tip of 4 μm O.D., the inner-filament was pulled to 160–180 nm O.D. In a typical backfilling, the solution migrates to the emitter tip to fill the tip and then the rest of the taper. During the process, there should be a transient moment when the solution initially arrives to partially fill the tip opening with a meniscus smaller than 4 μm. This type of inner-filament itself has been widely used to assist solution transport in capillaries for microelectrode, mass spectrometry, and patch clamp applications.^28^ However, the partially filled tip associated with this individual liquid channel has not been utilized.

Maintaining the partially filled state requires an equilibrium between solution supply and consumption. A setup composed of an alternating current discharge plasma source^29^ and an electrode pusher was built to provide a steady flow of charges to the emitter. Description of the setup and plasma discharge is detailed in Fig. S1–S5, ESI section 1 and 2.† The charge/ion transport to the tip of the capillary emitter was through the space external to the capillary, Fig. S6–S8, ESI section 3.† The sample solution in the emitter tip was readily ionized by the charge supply to maintain the partially filled meniscus. This mode of ionization is referred to as the sub-channel regime, to differentiate from electrospray ionization from a fully filled tip opening, which is called the main-channel regime. Interestingly, the sub-channel regime produced spray plumes that were narrower and sometimes barely observable, while the ion signal observed by mass spectrometer was continuous and intense, Video S1, ESI section 4.† The plasma source was 4–9 cm away from the ESI plume and did not produce APCI type ions.^30^ Both regimes ionized compounds to the same types of typical ESI ions (Table S1†), suggesting that charged droplets play a major role in the sub-channel regime.

Supplying charge using ions in the air was found to be critical for maintaining ESI from the sub-channel. ESI section 6† describes an alternative approach using DC high voltage applied to emitter with an external metal coating. Although solution delivery along the inner-filament was easily replicated, creating a continuous electrospray from the sub-channel was found to be challenging using DC voltage applied on the metal coating. Instead, pulsed electrospray from the main-channel was observed, Fig. S9.† The relative ion intensity of saccharide, indicating electrospray from a μm-scale meniscus, did not alter throughout the pulses. This suggests solution accumulation in the main-channel was necessary for adequate electrical contact. Increasing the voltage did not help with electric contact, but lead to discharge in air at 2.5 kV that suppressed ESI signal, Fig. S10.† These results suggest that electric contact *via* external metal coating was not as effective for charging the meniscus in the sub-channel.

**Fig. 1:**
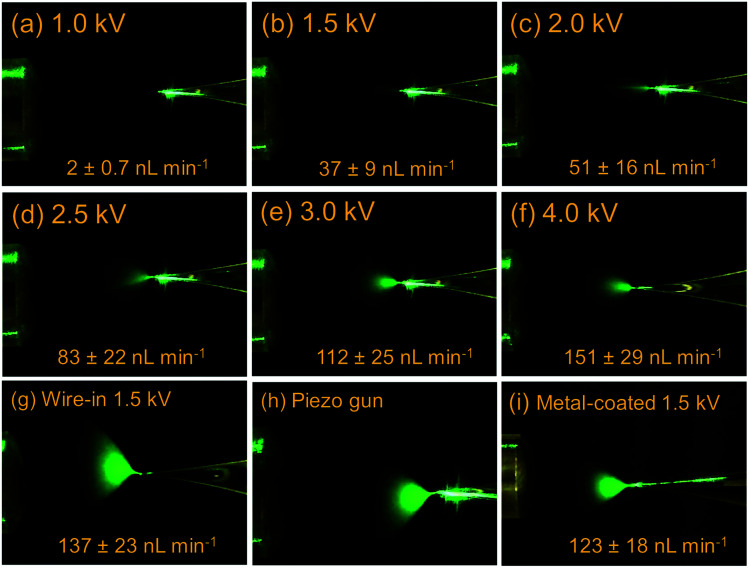
(a–f) Electrospray ionization plumes of a 26 μM MRFA solution (MeOH : H_2_O, 1 : 1) from a fully filled emitter tip under increasing pusher voltages. Electrospray plumes by (g) wire-in nanoESI, (h) relay-ESI triggered by a piezo gun, and (i) a metal-coated emitter. As a reference, the MS inlet shown in the figures has O.D. of 2.14 mm.

In this work, ionized air acted as a flexible “gas-phase electrode” that is always in contact with menisci associated with all flow conditions. It should be classified as a plasma–liquid interface, which has been used for providing electrode-free contact for bulk solutions.^31,32^ When the tip is fully filled, *i.e.* main-channel regime, the ionization mechanism was believed to be similar to the previously reported relay-ESI.^33^ However, a parameter space of higher dimension is provided by this work, which separately controls the solution flow, the charge supply, and the electric field. Fainter plumes with narrower spread were generated, Fig. 1. When the pusher electrode's voltage was increased from 1.0 kV (onset) to 4.0 kV, the spray plume gradually grew more intensive and looked brighter under laser illumination. The ESI flow rate increased from 2 nL min^−1^ to 150 nL min^−1^. The 150 nL min^−1^ at 4 kV was comparable with the flow rate of a 1.5 kV wire-in nanoESI. Yet, the different plumes suggest that smaller droplets were generated when using the gas phase electrode. The observed MS signal intensities increased by 2-fold when pusher voltage increased from 1.0–1.5 kV. Beyond 1.5 kV, the increase of signal intensity was less significant, Fig. S11.† In the following experiments, pusher voltages of 1.5–2.5 kV were adequate to spray solutions faster than what the capillary flow can supply. This would either lead to or maintain the sub-channel regime.

**Scheme 1:**
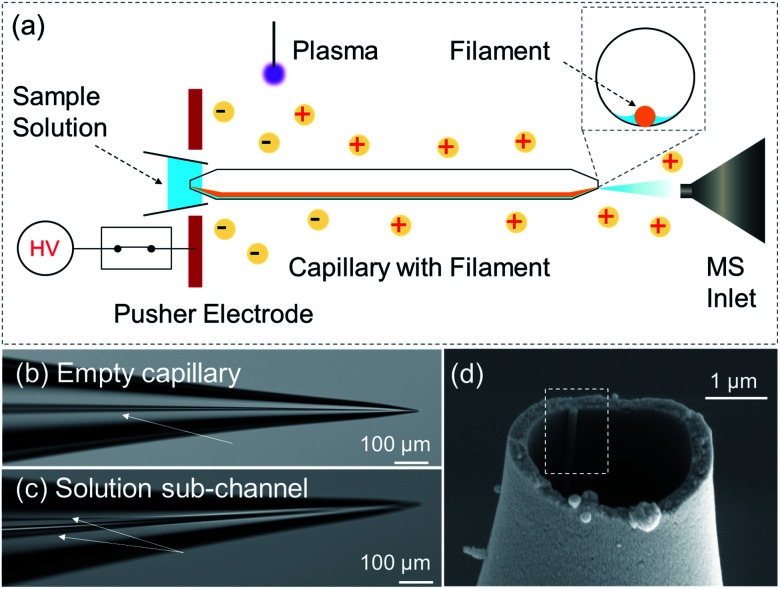
(a) Scheme of electrospray ionization directly from a sub-channel inside a capillary emitter. Sample solution is supplied to the emitter tip *via* the sub-channel from the back end. Pusher electrode guides plasma charges to triggers ESI at the emitter tip, which maintains the sub-channel meniscus. (b) Optical microscope images of the empty emitter, arrow indicates the inner filament. (c) Partially filled emitter tip, arrows indicate the edges of the solution sub-channel. (d) SEM image of the tip opening. The dashed rectangle highlights the inner filament which was pulled to 180 nm OD.

In the sub-channel regime, the electrospray flow is limited by the capillary flow in the sub-channel. The capillary flow is a passive liquid delivery phenomenon that becomes dominant at microscale and nanoscale.^34^ By arbitrarily adjusting the distance and volume of the reservoir solution, sub-channel flow rates ranging from 47 pL min^−1^ to 12 nL min^−1^ were achieved in this work, Fig. S12, ESI section 8 and 9.† Under these flow rates, the sub-channel was a solution layer along the inner filament, Scheme 1c. This solution layer had widths that are in the same order of magnitude as the inner-filament. In the taper region where the filament was pulled to gradually shrink to sizes undiscernible by optical microscope (160–180 nm by SEM), the width of the thin film also gradually shrinks to a size that is undiscernible. The sub-channel is an open channel that does not clog by itself. So, the picoflow ESI by nested-ESI in this work was as robust as nanoESI. It was maintained in a demonstration for almost 5 hours, Fig S13.† Compared with pulsed ESI methods,^16,33,35,36^ sub-channel ESI provides the direct current (DC) ion signal needed for the investigation of ionization performances.

**Fig. 2:**
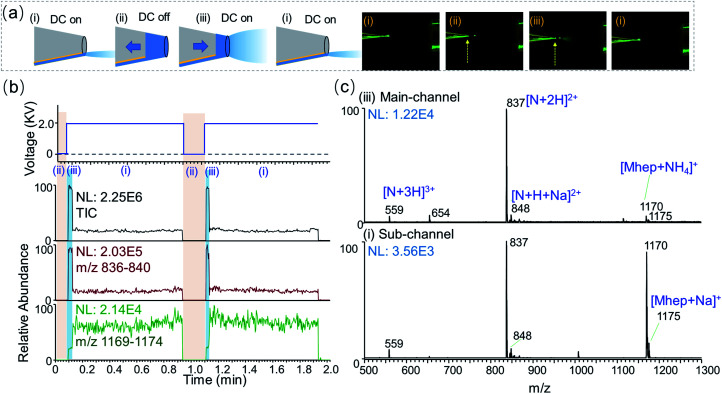
Alternation between sub-channel and main-channel regimes. A solution of 10 μM maltoheptaose (Mhep) and neurotensin (N) was analyzed. (a) Left: schemes showing electrospray from the sub-channel and the main-channel, respectively. The arrows indicate the solution being accumulated or consumed. Right: photos of emitter tip in front of the MS inlet. ESI plume of the main-channel regime was visible. The dashed arrows indicate the liquid–air boundary of the accumulated solution. (b) The DC voltage applied to the pusher electrode and the corresponding ion chronograms for total ion count, neurotensin, and maltoheptaose, respectively. (c) Mass spectra obtained in the main-channel (6.1 nL min^−1^) and sub-channel (530 pL min^−1^) regimes.

One unique feature of nested-ESI is that it allows rapid alternation between main-channel and sub-channel regimes in the same experiment. The pusher electrode provides instantaneous electronic control over the charge supply that is independent of the solution flow. As illustrated in Fig. 2, turning off the pusher voltage in the middle of a sub-channel regime instantaneously shut down the electrospray, allowing the capillary flow to gradually fill the emitter tip. Turning the pusher voltage back on initiated ESI from the main-channel. Once the solution accumulated in the main-channel was consumed, the ESI returned to the sub-channel regime. Transitioning from main-channel to sub-channel was accompanied by a change in the ESI plume that can lead to its “disappearance” from observation, Fig. 2a. By simply switching the pusher voltage on and off, a rapid alternation between the two regimes was achieved. To our best knowledge, this rapid alternation has not been demonstrated in previous low flow ESI researches, which required time-consuming flow stabilization or emitter swapping for different flow regimes.^9,10^ Rapid alternation using the same emitter and sample is advantageous when carrying out repeated comparison experiments. On-demand alternations using arbitrary time pattern also help to rule out potential contributions from other effects that would have their own time profiles.

Smaller initial charged droplets in the sub-channel regime were suggested by the optical observations during the alternation. Although ESI droplets could all evolve to become smaller *via* evaporation and fission, the total amount of charge is determined at the generation of initial charged droplets. Smaller-sized initial droplets generated by lower flow rates and smaller emitters were found to significantly improve the ionization efficiency of saccharide analytes, which are known to have poorer surface activities than peptides and are suppressed under the typical electrospray ionization conditions.^10,37^ In this work, the generation of smaller initial charged droplet by the sub-channel regime was confirmed by the ionization behavior of an equal molar mixture of maltoheptaose (Mhep) and neurotensin (N). In the main-channel regime, the signal intensity ratio between Mhep and neurotensin resemble those obtained using wire-in nanoESI. When alternating into the sub-channel regime, an abrupt decrease of peptide ion signal was observed in synchrony with (1) the disappearance of solution in the main-channel and (2) the dimming of ESI plume. This sudden change aligns with the proposed rapid transition between two distinct channels and associated menisci. The sub-channel meniscus at the tip is believed to also adhere to the nm-sized inner-filament at the tip. Rough estimations suggest that the cross-section of the sub-channel meniscus at the tip could be 200–2000 times smaller than that of the 4 μm main-channel.

One surprising result of the sub-channel regime ESI is the increase of absolute ion intensity of saccharides. In previous low flow ESI researches, enhanced ionization efficiency was obtained at the cost of decreasing (30-fold) absolute ion intensities.^10^ During the transitions into sub-channel regime, the peptide ion intensity and the total ion current (TIC) plummeted within 1 scan event, while the absolute ion intensity of Mhep did not decrease. To our surprise, the absolute intensity of Mhep would increase 2–5 folds, Fig. 2b and S14.† Plotting the Mhep intensities against the neurotensin intensities for all the experimental data points, it is evident that the sub-channel regime produced saccharide ion of much higher intensity, Fig. S15.† When the flow rate in the sub-channel was between 4–12 nL min^−1^, the relative Mhep intensity gradually increased from 20% to 50% with the decreasing neurotensin intensity. When the flow rate was lower than 4 nL min^−1^, the sub-channel regime gave predominantly equal responses (100% ± 10%) for Mhep and neurotensin. This equal response is in the character of that obtained by wire-in nanoESI using a 100 nm emitter tip, which had much lower absolute intensities. In one experiment, the flow rate of the main-channel was always higher than that of the sub-channel. However, the higher sub-channel regime flow rates (up to 12 nL min^−1^) in one experiment could exceed the lower main-channel regime flow rates (down to 2–5 nL min^−1^) in another. The data points in Fig. S15† indicate that the channel status is more important in determining the ionization behavior of analytes, and the flow rates of ESI regimes may overlap to some extent. It is worth noting that the ion source alternate into the sub-channel regime solely due to the variation of liquid meniscus, while the plasma and electric field were kept constant. Separation caused by electric field changes^30,38^ was not observed in the experiments of this work.

Enhanced absolute ion intensity in the sub-channel regime was also observed for a few other saccharides. For lighter molecular weight maltose in a mixture with a peptide angiotensin II, a similar trend was observed. Peak intensity ratios (maltose *m*/*z* 365: angiotensin II *m*/*z* 524) of 0.020 and 1.3 were observed in the main-channel and sub-channel regimes, respectively, Fig. S16.† For a mixture of turanose and *n*-octyl-glucopyranoside (OGP), a 10-fold increase of the relative ion intensity of turanose was observed in the sub-channel regime, Fig. S17.†

The sub-channel regime also enhances ionization efficiency for glycopeptide. Glycopeptide is a class of antimicrobials that are analyzed in high volumes in therapeutic drug monitoring.^39^ It is one example of the saccharide residues containing compounds produced by the many important glycosylation processes in cells.^40,41^ The hydrophilic saccharide residues usually reduce the ionization efficiencies of the analytes in ESI.^42^ For a solution mixture of vancomycin and angiotensin II, regime alternation brought a 1.9-fold increase of absolute ion intensity for the glycopeptide vancomycin, Fig. S18.† The increase of ion intensity of vancomycin in the sub-channel was less significant than the saccharides in previous cases. This is likely because its ionization in the main-channel regime was not as highly suppressed due to the presence of surface-active groups in its peptide backbone. The obvious change in ion intensity served as an indicator to pick out the peaks of the analyte that has hydrophilic moieties.

**Fig. 3:**
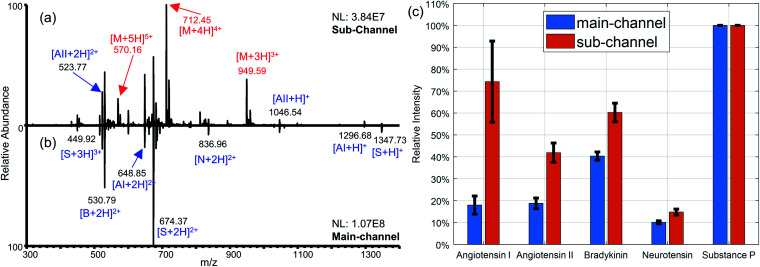
Equal concentration mixture of peptides (AI: angiotensin I, AII: angiotensin II, B: bradykinin, M: melittin, N: neurotensin, S: substance P) analyzed by nested-ESI. (a) Melittin was ionized with high efficiency to be the most intense peak in the sub-channel regime, while (b) significantly suppressed in the main-channel regime. The spectra were collected by LTQ-Orbitrap which gives higher normalized level (NL). (c) The intensities of the other peptides (angiotensin I : angiotensin II : bradykinin : neurotensin : substance P) normalized against that of substance P were 0.18 : 0.19 : 0.40 : 0.10 : 1.00 and 0.74 : 0.42 : 0.60 : 0.15 : 1.00, in the main-channel and sub-channel regimes respectively. Error bars represent one standard deviation of uncertainty.

Nested-ESI also exhibits different trends for more challenging analytes systems. Unlike saccharide–peptide mixtures,^10,12,43^ peptides have much less difference in surface activity between each other to produce significant different ionization efficiencies in lower flow rates. Smith *et al.* concluded in their picoelectrospray work that the relative ionization efficiencies of peptides were analyte-dependent and insensitive to flow rates in the range from 300 nL min^−1^ to 300 pL min^−1^. In particular, melittin had the least intensive peaks (<1%) under all flow rates.^9^ When analyzing a similar peptide mixture by nested-ESI, the main-channel regime had relative peak intensities similar to those in Smith's work. In the sub-channel regime, the intensity of melittin ions increased by two orders of magnitude and became the base peak (100%), Fig. 3. In nested-ESI, melittin behaved very much like the saccharides but with even larger enhancement. This is likely because it was more severely suppressed in the main-channel regime. For the rest of the peptides, their relative intensities were significantly more uniform in the sub-channel regime, Fig. 3b. It is worth noting that the flow rates of sub-channel regime here were 0.71–2.2 nL min^−1^, which is higher than the 0.4 nL min^−1^ in Smith's work. This indicates the smaller meniscus in the sub-channel regime is more critical than the lower flow rate for the observed ESI phenomena. Although an exact uniform response was not yet achieved for the peptide ions, the more uniform relative intensities obtained in the sub-channel regime of this work suggest that the observed ionization efficiency of peptide is not solely analyte-dependent as previously believed. Chemical and physical environments of charged droplets can significantly impact ionization efficiencies. Ways to manipulate the charged droplets is still worth exploring for a deeper understanding of the ionization process, which may lead to better and eventually uniform ion response.

**Fig. 4:**
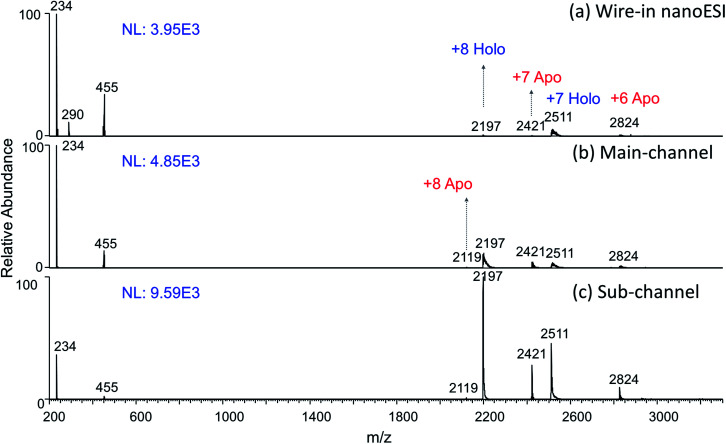
A sample of 10 μM myoglobin in 1 mM aqueous ammonium acetate being analyzed by (a) wire-in nanoESI at 40 nL min^−1^, as well as by the (b) main-channel regime at 5 nL min^−1^ and (c) sub-channel regime at 0.8 nL min^−1^ of nested-ESI. Ions of *m*/*z* 234, *m*/*z* 455 are from solvent background. Higher ionization efficiency and average charge states was obtained in the (b) and (c) while the native formations were preserved.

It was also found that peptides and proteins were ionized to higher averaged charge states (ACS) by the sub-channel regime. This trend is in accordance with the results by using smaller emitters,^44^ but opposite to those results by using the same emitter at lower flow rates.^45^ This again supports the presence and predominant roles of smaller ESI meniscus in the sub-channel regime. Additional effects from plasma ions are also suggested by the slightly higher charge states in the main-channel mode compared with those obtained by wire-in nanoESI, such as myoglobin in Fig. 4 and ubiquitin in Fig. S19.† Note that the shifts of the charged state were all among the typical charge states corresponding to the folded conformations. Native conformations, including the non-covalently bound heme group of myoglobin, were preserved. This suggests that plasma ions in this work did not infiltrate into the positively charged droplets to denature the proteins like what acid vapor would do.^46^ One explanation for the observed ACS shift is that the plasma produced protonated water and solvent cluster in the surrounding air. This would reduce proton transfer from analytes ions to neutral gas phase solvent clusters. This kind of charge retention^47,48^ would lead to the observed higher charge states without denaturing the proteins.

It is worth noting that higher ionization efficiencies were observed for proteins in nested-ESI, particularly the sub-channel regime. This effect is evident in both absolute ion intensity, and intensity relative to the solution background ions, Fig. 4. This effect is also evident when there was a minimal (23%) difference between the flow rates in the two regimes for cytochrome c, Fig. S20.† This is one more evidence confirming that the sub-channel meniscus is more critical than the flow rate for the observed effects. Considering that proteins were ionized by the charge residue model and tend to stay in the droplets during ESI evolution, the observed signal enhancements in sub-channel regime are consistent with those of saccharides. Ionized air was found to induce more evenly split during the coulombic fission of μm-sized charged droplets.^49^ If this trend extend to sub-μm droplets, the ionized air produced by the plasma ions in this work would lead to a faster elimination of large residue droplets. This would be beneficial for evaporating droplets in the charge residue model, which could explain the significantly enhanced ionization efficiency for the proteins. One typical application of the smaller charged droplets is the reduction of salt clusters and non-specific adducts. Besides achieving these effects in the sub-channel regime, comparing the two sets of data from the rapid regime alternation allowed facile identification of non-specific adduct ions, Fig. S21, ESI section 13.† Protein's higher ionization efficiency in the sub-channel regime is also evident for this salt-containing sample solution.

**Fig. 5:**
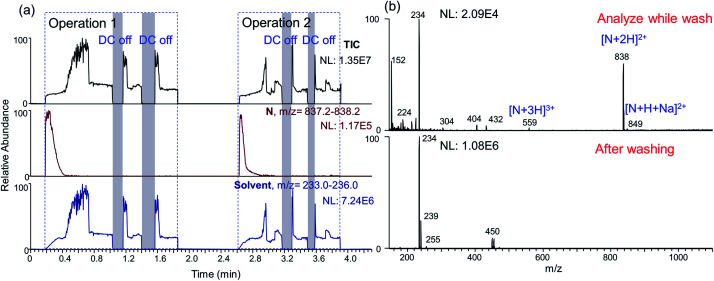
Sample solution was aspirated into an emitter tip and analyzed by nested-ESI. While the sample solution (10 μM neurotensin) was electrosprayed by the main-channel, blank solution in the sub-channel washed the emitter tip. (a) TIC and EICs show two operations. (b) Mass spectra show that the analyte signal during the wash, and the solvent background ions after the wash.

Besides enabling on-demand alternation between different regimes, the nested-ESI also provides a platform to online wash the emitter tip when needed. Direct nanoESI analysis is indispensable for trace samples analysis,^50,51^ shotgun lipidomics,^52^ and cell analysis.^53,54^ One limitation of direct nanoESI analysis is the carryover contamination. For nested-ESI, carryover contamination is localized in a much smaller region due to the largely empty emitter. Another unique feature of nested-ESI is that the emitter tip can draw solutions from both the sub-channel and the front end. By using the front end to load sample solution, while supplying blank solvent from the sub-channel, the emitter was washed during the nanoESI analysis (ESI section 14†). Illustrated in Fig. S22,† an emitter in the sub-channel regime (2 nL min^−1^) was taken off from the platform for manual sampling. The manual operation took 15 s, in which time the main-channel was filled by 0.5 nL. Then, dipping this emitter tip into a sample solution for 9–12 s aspirated roughly 5 nL sample solution. Mounting the emitter back to the platform took another 30 s, during which time another 1 nL blank solvent would accumulate in the main-channel. Then the loaded sample was analyzed by nested-ESI. The ionization was initially in the main-channel regime, during which the inner surface was continuously washed by the flow of blank solvent from the sub-channel. 10 μM analytes were effectively washed before ESI enters sub-channel regime, as revealed by the full scan (Fig. 5) and MS/MS (Fig. S23†). Compared with dipping emitter tip into bulk quantities of blank solvent (Fig. S24 and S25†), solvent flow in sub-channel is effective for washing the emitter tip. Regime alternation is necessary to empty the emitter tip for the next operation of sampling and analysis. It may also be adopted when repeated washing of the tip region is needed.

## Conclusion

In this work, a dynamic nanoscale to microscale liquid layer adhered to local structures inside a capillary emitter was maintained for electrospray ionization. Charges supplied from the air ionize the sample solution directly from the meniscus of the liquid layer (sub-channel), before the tip opening of the emitter may be filled (main-channel). The two nested channels allow an emitter tip to accommodate a wider range of electrospray flow rates. In the sub-channel regime, the partially empty emitter tip, the fainter ESI plume, the enhanced saccharide ion signal and the higher average charge states all suggest that smaller initial charged droplets were produced from its meniscus.

Separated controls over the exterior charge supply and interior solution flow allow the ESI to alternate between sub-channel and main-channel regimes on-demand. For the first time, this rapid alternation allowed repeated and reliable comparisons between ESI regimes. The sub-channel regime enhanced the ionization efficiency of hydrophilic compounds and proteins to much higher magnitudes. Non-specific adduct was reduced and identified by regime alternation. The nested two channels also allowed facile washing of the emitter tip during sample analyses.

This work provides a new avenue to generate ESI with lower flow rates and smaller initial charged droplets. Particularly encouraging is the trend toward a more uniform ion response that was not observed in previous picoelectrospray study. This suggests the observed ion response is not just analyte-dependent and there is much more to be explored for the generation, manipulation and better understanding of ESI droplets.

## Conflicts of interest

There are no conflicts to declare.

## Supplementary Material

SC-012-D0SC06221A-s001

SC-012-D0SC06221A-s002
