# Supplementary material for: Nested-channel for on-demand alternation between electrospray ionization regimes
Source: Chem Sci. 2020 Dec 22;12(5):1907–14. doi: 10.1039/d0sc06221a (PMC8179270; doi:10.1039/d0sc06221a)
Supplement: SC-012-D0SC06221A-s002 [file SC-012-D0SC06221A-s002.pdf]

Electronic Supplementary Material (ESI) for Chemical Science.
This journal is © The Royal Society of Chemistry 2021

Supporting Information

Nested-channel for On-demand Alternation between Electrospray Ionization Regimes

Mengtian Li,[a] Huishan Li,[a] Nicholas R. Allen,[a] Taoqing Wang,[a] Linfan Li,[b] Jae Schwartz,[b] Anyin Li*[a]

[a] Department of Chemistry, University of New Hampshire, 23 Academic Way, Durham, NH 03824

[b] Thermo Fisher Scientific, 355 River Oaks Pkwy, San Jose, CA 95134

*Corresponding authors: Anyin.Li@unh.edu

Table of Contents:

1. The nested-ESI setup and MS instruments

Figure S1. A prototype of the setup.

Figure S2. The position of plasma, pusher electrode, and capillary emitter.

2. The plasma charge supply

Figure S3. Input voltage for PT discharge plasma.

Figure S4. Driving frequency of PT discharge plasma.

Figure S5. Ions generated by the discharge plasma in air.

3. Charge transport to the meniscus

Figure S6. Sealed vs. open (back end) capillary emitters.

Figure S7. Shielding charge on the halfway shuts down the ESI.

Figure S8. Grounding emitter tip shuts down the ESI.

4. Rapid alternation of ESI regimes (Video S1)

5. Reagents, samples and types of generated ions

Table S1. Types of molecular ions generated by nested-channel electrospray ionization.

6. Sub-channel and the "gas-phase electrode"

Figure S9. Pulsed electrospray using metal-coated emitter with sub-channel and DC power supply.

Figure S10. The effect of increasing DC voltage for coated externally emitters.

7. MS signal of MRFA for different DC voltage

Figure 11. Relative intensity of MRFA (m/z=524) for different DC voltage.

8. Methods for sample loading and flow rate control

Figure S12. Three methods for loading sample solution and flow rate control.

9. Flow Rate Measurements

10. Stability of nested-ESI

Figure S13. Continuous nested-ESI analysis for almost 5 hours with an initial flow rate of 0.4 nL min^{-1}.

11. Rapid alternation of flow regimes

Figure S14. The EIC data points during typical transitions from main-channel regime to the sub-channel regime.

Figure S15. Comparing regime alternation with wire-in nanoESI and picoESI.

12. MS spectra in the main-channel and sub-channel regimes

Figure S16. Angiotensin II and maltose.

Figure S17. Turanose and n-octyl-glucopyranoside (OGP).

Figure S18. Vancomycin and angiotensin II (AII)

Figure S19. Ubiquitin, wire-in vs. regime alternation

Figure S20. Cytochrome c, regime alternation

13. Application I: desalting and decreasing non-specifically adducting by alternating main-channel and sub-channel

Figure S21. TIC, EICs and MS spectra of cytochrome c in salt solution.

14. Application II: analyzing and washing online through sub-channel

Figure S22. Analysis-while-washing by using the two channels of nested-ESI.

Figure S23. TIC, EICs, mass spectra and tandem mass spectra obtained using analysis while washing by nested-ESI.

Figure S24. Schematic of an experiment in which blank solvent was added from the front end as an alternative way to wash the emitter tip.

Figure S25. TIC, EICs and mass spectra of control experiment by using capillary with filament.

1. The nested-ESI setup and MS instruments

The setup is illustrated in Figure S1 and S2. Plasma ion source, pusher electrode, and filament containing emitter tip are three core components for nested-ESI.

The plasma ions were generated by using a piezoelectric transformer (53×7.5×2.6 mm, INC model SMSTF68P10S9, Steiner & Martins), with the advantages of compact size, low power consumption and safety, and operating in atmospheric air, as shown in the inner photo of Figure S1. The piezoelectric transformer was operated by supplying an input voltage (5-25 V, Powertron Model 500A; Industrial Test Equipment Co. Inc., Port Washington, NY, USA) triggered by a sine waveform. Plasma discharge was readily generated at the tip of the output electrode under ambient conditions. The faint plasma may be observed by naked eye. A pusher electrode (44×44 mm) was placed behind the capillary emitter to create an auxiliary electric field, which pushed positive or negative plasma ions to the capillary emitter.

Typical alignment parameters for plasma ion source, pusher electrode and capillary emitter were listed in Figure S2.

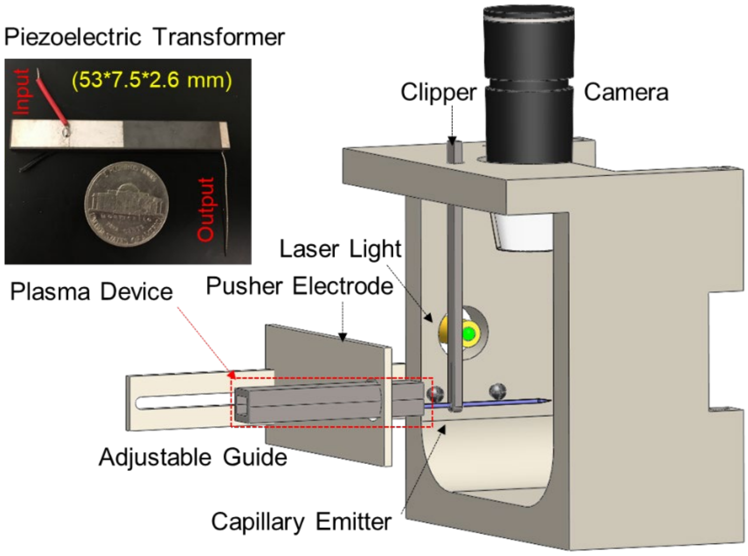

Figure S1. A prototype of the setup, the inset photo shows the piezoelectric transformer.

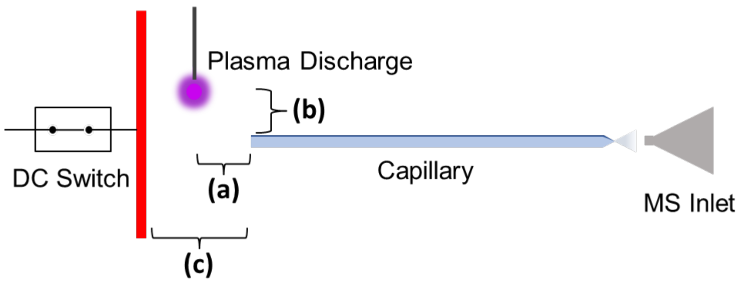

**Discharge plasma conditions**	**Parameters**
Plasma to capillary **(a)**	-3-3 mm
Parallel distance **(b)**	0-5 mm
Pusher electrode to the capillary **(c)**	-3-15 mm

Figure S2. The positions of plasma, pusher electrode, and capillary emitter.

An LTQ-XL linear ion trap and an LTQ velos Orbitrap mass spectrometer (Thermo Scientific, San Jose, CA) were used in the experiments. Unless mentioned otherwise, the LTQ-XL was operated with following parameters: inlet temperature 125 ºC, inlet capillary voltage 9 V, tube lens voltage 100 V. LTQ velos Orbitrap was operated under the conditions: inlet temperature 125 ºC, S-lens RF level 55%. All mass spectra were recorded as peak profiles with 1 microscan and 10 ms maximum injection time. Isolation window of 1.5 m/z and arbitrary collision energy of 15-45 were used for MS/MS experiments.

A micropipette puller (model P-1000, Sutter Instrument, CA) was used for pulling emitters. Borosilicate glass capillaries, with and without filament, (o.d., 1.5 mm; i.d., 0.86 mm; BF 150-86-10 and B 150-86-10) was used. Metal-coated glass emitters were purchased from New Objective (Pico Tip Emitter, BG 12-94-4-CE-20, tip 4±1 µm, Woburn, MA). The emitter tips were checked by bright-field microscopy (Olympus IX73), as well as measured by a field emission scanning electron microscopy (TESCAN LYRA3) after sputter coating a 20 nm layer of Pd/Au. A micro butane torch was used to seal the proximal end of emitters when needed.

2. The plasma charge supply

Figure S3 shows the input for the piezoelectric (PT) discharge plasma (15 V at 67.3 kHz). The actual resonance frequency of the PT slightly alters from crystal to crystal. So, the effects of driving frequency for the discharge status and input current need to be characterized, as shown in Figure S4. The input current at the maximum was 64 mA, corresponding to a brightest corona discharge at the tail end of the output electrode (Figure S4a). So, 67.3 kHz was finally chosen as the optimized condition. Under this condition, the power consumption was ~960 mW.

At this condition, the piezoelectric discharge plasma generates adequate cations and anions continuously, as can be observed by MS in the positive and negative modes (Figure S5). Typical air plasma ions, such as protonated water clusters $[(H_2O)_nH]^+$ and anions (O_2^-, OH^-, NO_2^-), were generated.

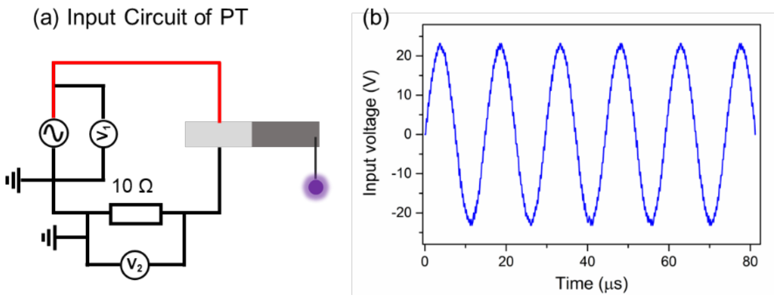

Figure S3. Input voltage for PT discharge plasma. **(a)** The input circuit diagram for input current (by meter V_2) and **(b)** the measured input voltage (by meter V_1) for the piezoelectric transformer (15 V, sine at 67.3 kHz).

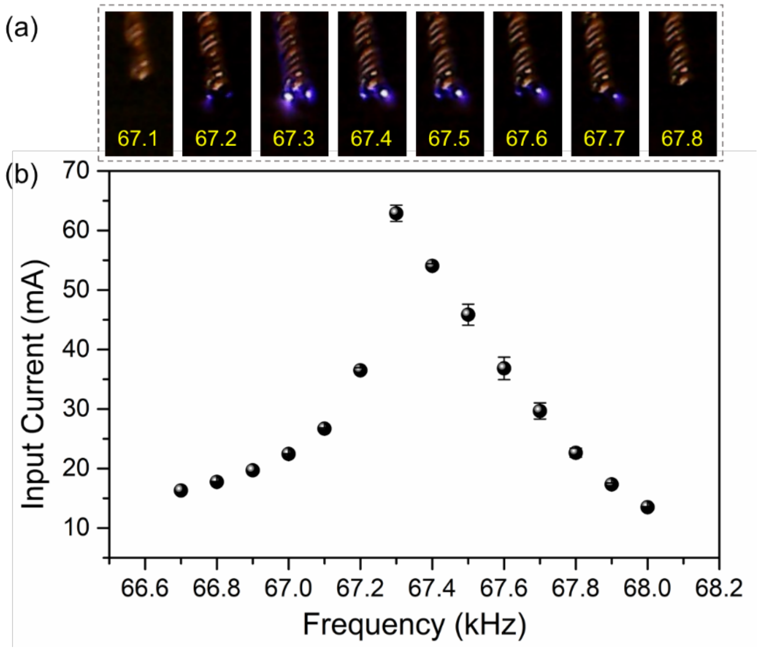

Figure S4. Driving frequency of PT discharge plasma. **(a)** Photos of plasma discharge. The diameter of the wire electrode (as shown in the photo) is 0.25 mm. The bright blue spots at the lower end of the output wire electrode are discharge plasma. **(b)** Input current vs. driving frequency. The input current was calculated using the voltage across a resistor (10 Ω), measured by meter V_2 in the circuit shown in Figure S3(a).

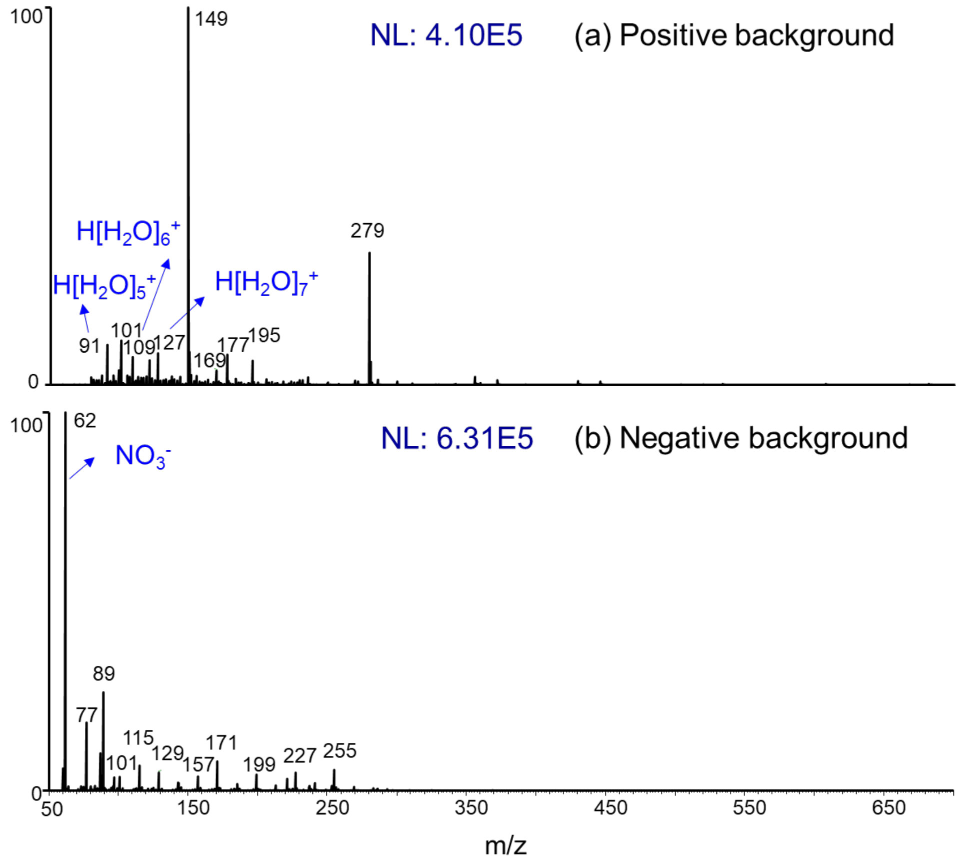

Figure S5. Ions generated by the discharge plasma in air, observed by MS in **(a)** positive and **(b)** negative modes. The discharge plasma was placed 5-10 mm away from the MS inlet.

3. Charge transport to the meniscus

Several experiments were designed to investigate how charges were transported from the plasma source to the ESI meniscus.

Firstly, capillary emitter with a fire-sealed proximal end was tested. As shown in Figure S6, electrospray from the sealed capillary emitter had no significant difference with that from the emitter with open proximal end. This suggests that the channels inside the capillary emitter are not needed for charge/ion transportation.

Secondly, two experiments further designed to verify the transportation from the surface and/or the space out of capillary, as shown in Figure S7. Cu tape wrapped around the middle section of the capillary was not able to shut down the electrospray by grounding. However, a Cu film (100×100 mm) placed perpendicularly to the capillary was able to effectively shut down the electrospray. These results suggest that the exterior space is critical for the charge transport. Fig. S7b also confirms that the

inner channels of the emitters are not responsible for charge transport.

Thirdly, grounding the emitter tip was found to effectively shut down the ESI (Figure S8), suggesting charges build up at the tip is critical for the ESI. Based all these results, it is concluded that the charges were transported via the exterior space surrounding capillary to the tip of the emitter, which leads to electrospray.

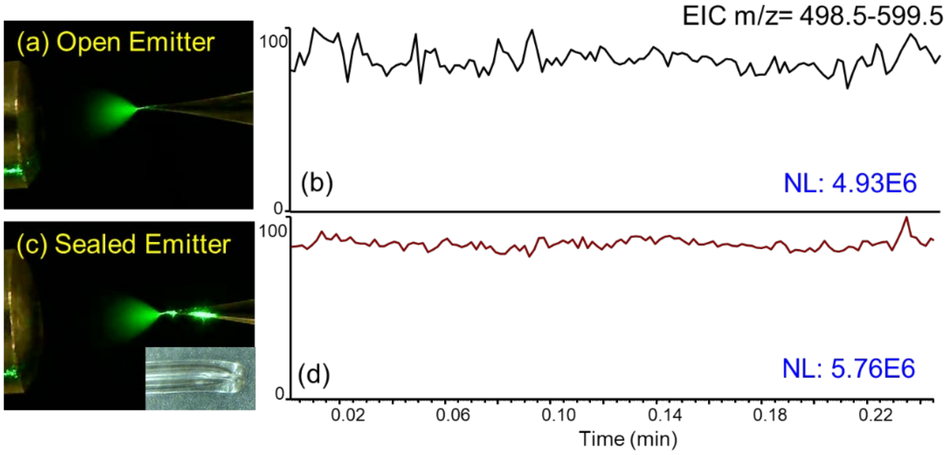

Figure S6. Spray plume and EIC of the PFOS analyte (m/z=498.5-499.5) for **(a, b)** an open proximal end emitter, and **(c, d)** sealed proximal end (inset) emitters. Experimental conditions: perfluorooctanesulfonic acid (PFOS), 1 µg mL^{-1} in mixture of water and methanol (v:v, 1:1); negative mode.

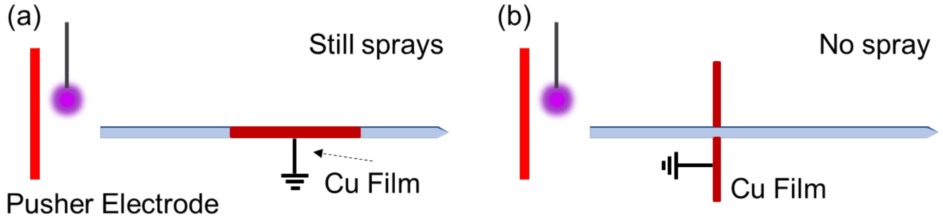

Figure S7. **(a)** Grounding the surface of middle section of emitter did not shut down the nested-ESI spray plume and ion signal. This suggests that the charges were transported through the exterior space. **(b)** A grounded shield (10×10 cm diameter) placed in the middle section of the emitter effectively shut down the nested-ESI spray plume and ion signal. Note the back end of the emitters are open, indicating that the charges were not transported through the channels inside the emitters.

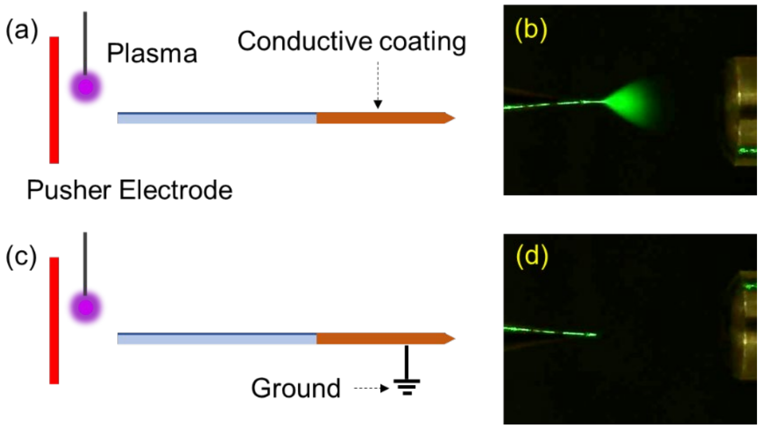

Figure S8. When a metal-coated capillary emitter was used, **(a, b)** gas-phase electrode generated electrospray. **(c, d)** Grounding the metal-coated tip effectively shut down the ESI.

4. Rapid alternation of ESI regimes

Video S1 shows the rapid alternation of ESI regimes by turning the pusher voltage on and off. The on and off was controlled by setting voltage to 0 or 1.5 kV using the user software of the mass spectrometer. When the voltage was set to 0, the spray was stopped, and the solution started to accumulate in the main channel (7-14 s). Right after turning the pusher voltage back to 1.5 kV, a bright plume of ESI droplets was generated, until the solution in the main-channel was consumed (14-17 s). After that the ESI entered sub-channel mode, which is characterized by an "empty" emitter tip, fainter ESI plume, and higher relative ion intensity for saccharide (0-7 s, 17-19 s).

5. Reagents and Samples and types of generated ions

Methanol (HPLC grade), water (HPLC grade), ammonium acetate, sodium chloride, dopamine hydrochloride, ethidium bromide, (±) propranolol hydrochloride, 3-nitrophenol, 4-nitrophenol, caffeine, octyl-D-glucopyranoside, 1,4-maltose monohydrate, D-(+)-turanose, maltoheptaose, L-methionine, L-methionine sulfoxide, glutamic acid, glutamic acid-$^{13}C_5$, ^{15}N, MRFA (Met-Arg-Phe-Ala acetate salt), myoglobin (equine heart), angiotensin I, angiotensin II, bradykinin, neurotensin, substance P, melittin, cytochrome c (equine heart), and neurotensin were purchased from Sigma-Aldrich (St. Louis, MO, USA). Vancomycin were purchased from Fisher Scientific company. Standard stock solutions of cocaine (1.0 mg mL^{-1} in acetonitrile) and trinitrotoluene (1000 µg mL^{-1} in Methanol and acetonitrile (v:v,1:1)) were

purchased from AccuStandard Inc. (New Haven, CT, USA). Perfluorinated Compounds (PFCs) were purchased from Wellington Laboratories Inc. (Guelph, Ontario, Canada). MRFA, myoglobin, angiotensin II and cytochrome c were prepared in aqueous ammonium acetate or sodium chloride solutions. Maltoheptaose and neurotensin were in the mixture of methanol and 10 mM aqueous ammonium acetate solution (v:v, 1:1). Samples without special note were prepared to the target concentrations by serial dilution using methanol and water (v:v, 1:1).

Table S1. Types of ions generated by nested-ESI for various compounds.

**Compounds**	MS Modes	Observed Analyte Ion
PFCs mixtures	neg	$[M-H]^-$
Nitrophenol	neg	$[M-H]^-$
Ethidium	pos	$[M]^+$
Dopamine	pos	$[M+H]^+$
Cocaine	pos	$[M+H]^+$
Caffeine	pos	$[M+H]^+$
Propranolol	pos	$[M+H]^+$
1,4-Maltose	neg/pos	$[M+Cl]^-$/ $[M+NH_4]^+$, $[M+Na]^+$
Turanose	pos	$[M+Na]^+$
Maltoheptaose	pos	$[M+NH_4]^+$, $[M+Na]^+$
Octyl D-glucopyranoside	pos	$[M+Na]^+$
Glutamic acid	neg	$[M-H]^-$
Methionine	neg/pos	$[M-H]^-$/$[M+H]^+$
Methionine Sulfoxide	neg/pos	$[M-H]^-$/$[M+H]^+$
MRFA	pos	$[M+H]^+$
Angiotensin I and II	pos	$[M+2H]^{2+}$, $[M+H+Na]^{2+}$, $[M+2Na]^{2+}$, $[M+H]^+$, $[M+Na]^+$
Bradykinin	pos	$[M+2H]^{2+}$, $[M+H+Na]^{2+}$, $[M+2Na]^{2+}$, $[M+H]^+$, $[M+Na]^+$
Melittin	pos	$[M+5H]^{5+}$, $[M+4H]^{4+}$, $[M+3H]^{3+}$
Neurotensin	pos	$[M+3H]^{3+}$, $[M+2H]^{2+}$, $[M+H+Na]^{2+}$, $[M+2Na]^{2+}$, $[M+H]^+$
Substance P	pos	$[M+3H]^{3+}$, $[M+2H]^{2+}$, $[M+H+Na]^{2+}$, $[M+2Na]^{2+}$, $[M+H]^+$, $[M+Na]^+$
Vancomycin	pos	$[M+2H]^{2+}$, $[M+H+Na]^{2+}$, $[2M+3H]^{3+}$, $[M+H]^+$, $[M+Na]^+$

6. Sub-channel and the "gas-phase electrode"

The gas-phase electrode (plasma ions + pusher electrode) was more effective than conventional voltage supplies in charging small meniscus. Here, applying DC voltage to metal-coated emitter tip was tried. As an unsuccessful attempt in triggering ESI from the sub-channel, it serves as a comparison to illustrate the effective charging mechanism of the gas-phase electrode.

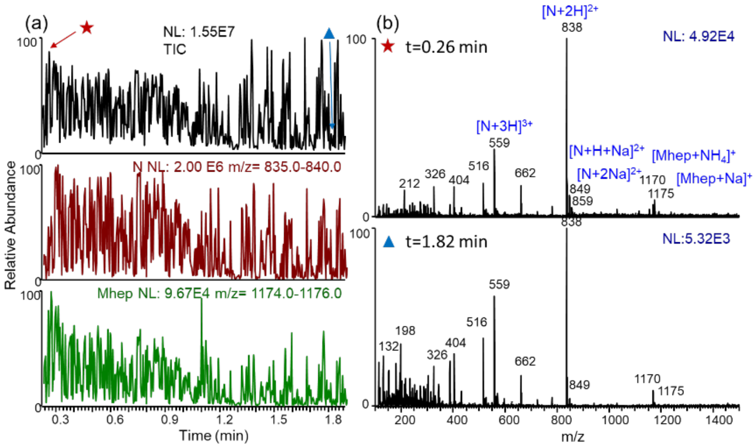

Figure S9. Apply a DC voltage (1.5 kV) to the metal-coated emitters when trying to ionize solution from its sub-channel. The pulsating electrospray was obtained. **(a)** TIC and EICs of maltoheptaose (Mhep) and neurotensin (N); **(b)** mass spectra corresponding to time points of higher and lower total ion current. Despite a 10-time difference in ion current, the intensity of the Mhep relative to that of neurotensin did not alter, suggesting the sizes of initial charged droplets were similar at the two timepoints. Experimental conditions: 10 μM maltoheptaose and neurotensin in mixture of methanol and 10 mM ammonium acetate aqueous solution (v:v, 1:1); applied DC voltage, 1.5 kV.

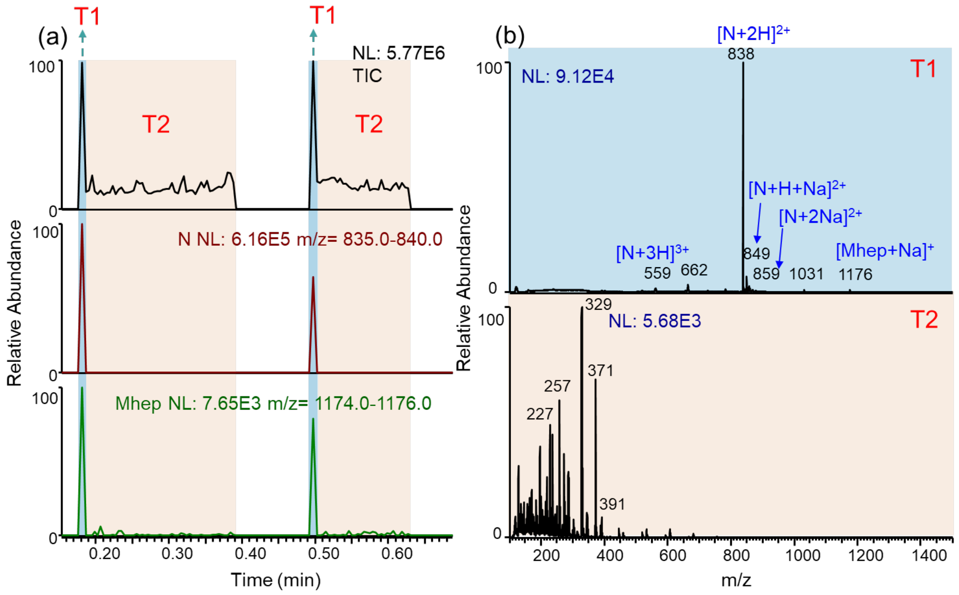

Figure S10. Apply a higher DC voltage (2.5 kV) to the metal-coated emitters when trying to ionize solution from its sub-channel. **(a)** TIC and EICs of maltoheptaose (Mhep) and neurotensin (N); **(b)** mass spectra for the corresponding time ranges. The results suggest air breakdown after a transient moment of ESI. The air breakdown terminated the typical ESI signals of the analytes. Experimental conditions: 10 µM maltoheptaose and neurotensin in mixture of methanol and 10 mM ammonium acetate aqueous solution (v:v, 1:1).

7. MS signal of MRFA for different DC voltage

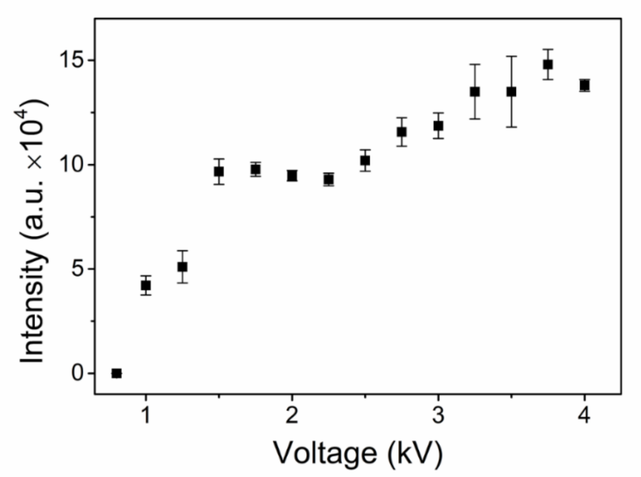

Figure S11. Relative intensity of MRFA (m/z=524) for different DC voltages. Experimental conditions: analytes, 26 µM MRFA in MeOH and water (v:v, 1:1).

8. Methods for sample loading and flow rate control

In this study, three methods were used for sample loading and approximate control of flow rates in the experiments.

Loading method #1 is to load > 5 μL sample solution into emitter tip, like what is commonly done for wire-in nanoESI. Flow rate control was realized by using different pusher voltages. The ESI in main-channel regime and the flow rates achieved using this method were in the range of 2-151 $nL \cdot min^{-1}$. The flow rate was measured by weight difference (balance) over long spray times.

Loading method #2 is to load sample solution into the proximal end of capillary emitter. The total lengths of the capillary emitters were 3-10 cm. A 4 μm tip opening with ~5 mm taper was heat-pulled at the distal end of these emitters. Typically, < 2 μL sample solution was loaded at the proximal end, which migrated automatically to the tip as a result of capillary action (Figure S12(b)). The solution migrated through the sub-channel along the inner filament. Migration flow rates ranging from 0.4 to 12 nL/min were achieved in the sub-channel using different emitter body lengths and loaded volumes. Typically, it takes 15-30 seconds for the sample solution to first reach the tip opening. Generally, lower flow rates were obtained using lower sample volume, longer capillary body. In this manuscript, this loading method was most widely used for the regime alternation experiments.

Loading method #3 is to supply sample solution to the heated-pulled proximal end of capillary emitter. A long capillary emitter (>80 mm) was pulled to sharp tips at both the proximal and distal ends. A large (~100 μL) drop of sample solution in a pipette tip was controlled by a linear motor (LinMot USA Inc.) to be in contact with the proximal end at desired frequency, Figure S12(c). Typically, it takes 2-5 minutes for the sample solution to first reach the tip opening. The linear motor moved back and forth in programmed patterns to control the contact time of the emitter tip to the solution. Tiny volumes of solutions were loaded to the proximal end in short pulses (<1 s) in adjustable duty cycles, typically set at 10%. The short pulses were found to be regulated into constant flow after migrating along the capillary body. Generally, shorter contact time and longer capillary body will give lower flow rates. This more complicated sample loading method was used to achieve the lower sub-channel flow rates in the range of 47-500 pL/min.

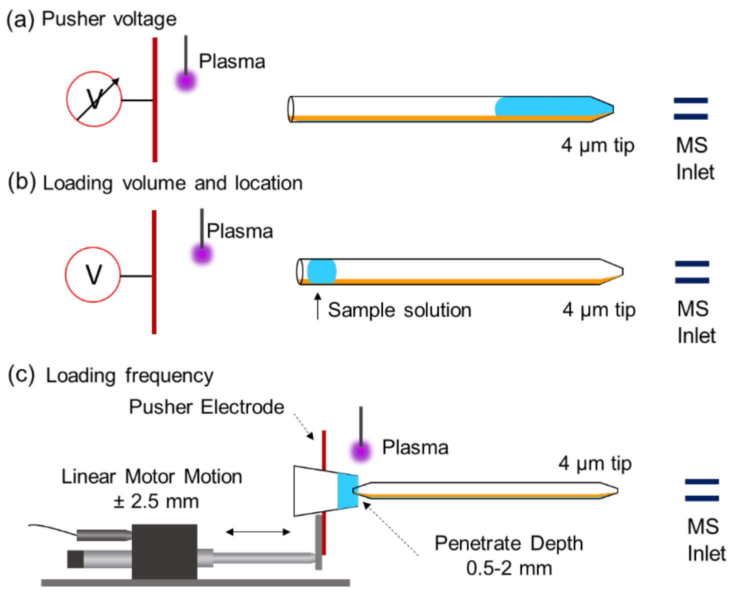

Figure S12. Three methods for sample introduction and flow control. **(a)** Solution was loaded to the emitter tip and the different flow rate was obtained by using different pusher voltages. **(b)** A drop of solution was loaded into the proximal end of capillary and migrated to the tip through the sub-channel. This method was used in most of regime alternation. **(c)** Solution was periodically supplied to the proximal end which was also pulled to a sharp tip.

9. Flow Rate Measurements

In this work, the flow rates of the ESI were determined using one of the following two methods.

Measurement method #1 is based on gravimetric analysis of the capillary emitter before and after spraying for a period. Given the spray time, the weight lost, and the density of the solution, the flow rate can be determined. The weight measurements were carried out using a Mettler Toledo MX5 microbalance (Mettler-Toledo, Columbus, OH; repeatability reported by manufacturer is ±0.8-0.9 μg). In our work, the total weight of capillary emitters typically ranged 0.134823-0.147074 gram. Standard deviations ranging 0.5-3 μg were obtained when weighing capillary emitters for 3 times in the experiments. The standard deviations before ($\boldsymbol{e_1}$) and after ($\boldsymbol{e_2}$) electrospray plugged into the equation $\boldsymbol{e} = \sqrt{\boldsymbol{e}_1^2 + \boldsymbol{e}_2^2}$ to calculate the propagated error $\boldsymbol{e}$. This propagated weight error $\boldsymbol{e}$ was divided by solution density and spray time obtain the flow rate error in each experiment. In one experiment, the standard deviations before and after spraying were 1.4×10^{-6} g and 2.1×10^{-6} g. Divided by the solvent density (0.927 g/mL

for methanol: water 1:1) and the 30 min spray time, a measurement error of 91 pL/min was obtained. This is the error for individual measurements, which controlled to be less than 1/3 of the flow rate by using long spray time if needed. For flow rates reported in Figure 1, the standard deviations of three replicate measurements were used as the errors. The replicate errors were generally larger than the individual measurement errors, due to unavoidable alignment variations of the three replicates, which is analogous to those of wire-in nanoESI.

Measurement method #2 is based on volume of solution accumulated in the tip emitter over time. This method was used when nested-ESI was alternated between sub-channel and main-channel regimes. When sub-channel ESI is equilibrated, solution flow rate in the sub-channel is approximately equal to the electrospray consumption rate. Temporarily shutting down the electrospray, solution will be accumulated in the emitter tip. Assuming the solution flow rate is constant in the first about 0.5 min of accumulation, accumulated volume over time will allow the calculation of flow rate through sub-channel. Likewise, flow rates for the main-channel regimes was calculated by how fast the accumulated solution was consumed and by including the sub-channel flow rate. In the experiments, videos were taken using a camera at 30 frame per second. Lengths in the video were calculated using a known object, 2.14 mm/228 pixels. The volume was calculated by measuring the height (h) and radius ($r = kh$ for a fixed cone shape) of the cone shaped solution. This calculated volume by $V = \frac{1}{3}\pi k^2 h^3$, was then divided by the time elapsed to yield the flow rate. In one experiment, the length of the accumulated solution was 11 pixels, giving a calculated volume of 9.5 pL. For a spray time of 0.17 min, this corresponds to a flow rate of 56 pL/min. The error in this flow rate was estimated using the potential error brought by miscounting 1 pixel during the volume calculation. 1 pixel per 11 pixel corresponds to a relative error of 9.1% for h. Considering the $V = \frac{1}{3}\pi k^2 h^3$ equation, the propagated error for the volume would be 27%. Relative error in time measurements, estimated based on supposedly miscounting one frame, are ~0.8% and always at least one order of magnitude smaller and thus omitted. In another experiment, the measured length of the bulk solution was 49 pixels, corresponding to a volume of 0.26 nL. For a spray time of 0.16 min, this corresponds to a flow rate of 1.6 nL/min. Propagated error from ±1 pixel would be 6%.

10. Stability of nested-ESI

The robustness of nested-ESI was exemplified in multiple experiment that spray more than 3 hours. An initial flow rate was measured by regime alternation and then the setup was left untouched. In one experiment, nested-ESI was set up at 0.4 nL min^{-1} and then sprayed continuously for almost 5 hours, shown in Figure S13. The initial increase of TIC and EICs after 5 minutes was likely due to the fluctuation of flow rate of the system. Despite signal fluctuations, the TIC and EICs indicate that there was no separation effect inside the channels caused by the external plasma and electric fields. Note that at 185 min, the flow rate decreased because a particle accidentally entered the emitter. Interestingly, this did not lead to a complete clogging of the emitter. Instead, it was slowly washed down the sub-channel and then ejected by ESI. After that, the flow and ion signals quickly recovered.

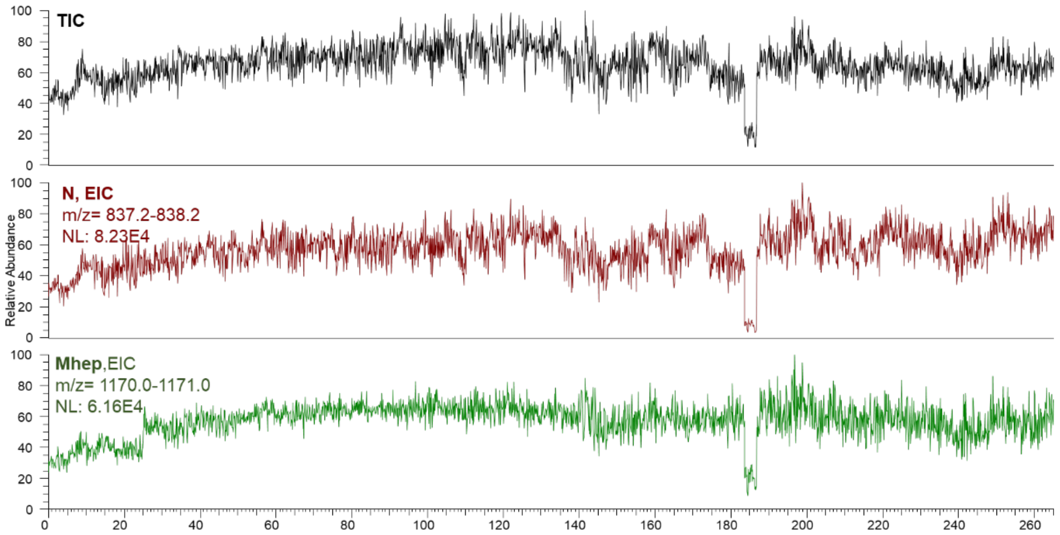

Figure S13. Continuously nested electrospray ionization for almost 5 hours with an initial flow rate of 0.4 nL min^{-1}. Sample being used was 10 µM maltoheptaose and neurotensin in a mixture of methanol and 10 mM ammonium acetate aqueous solution (v:v, 1:1). At 185 min, the flow rate decreased because a particle accidentally entered the emitter. Interestingly, this did not lead to a complete clogging of the emitter.

11. Alternation of ESI regimes on-demand

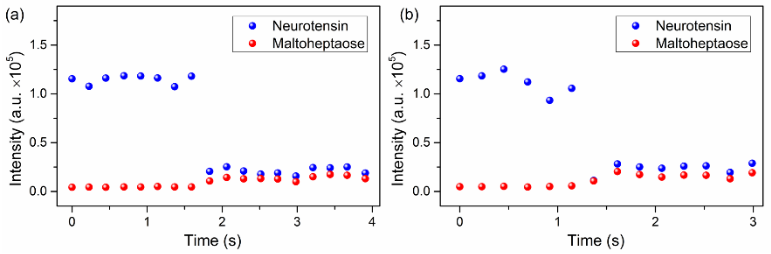

Figure S14. The EIC data points during typical transitions from main-channel regime to the sub-channel regime. Neurotensin's ion intensity plummets rapidly within 1 scan event, the increase of saccharide's ion intensity jumped in 1-2 scan events in our experiments. Mass range of neurotensin, m/z=837.2-838.2; mass range of maltoheptaose, m/z=1169.5-1170.5. The 7th points of N and Mhep in Figure S14(b) were overlapped. Experimental conditions: 10 µM maltoheptaose and neurotensin in mixture of methanol and 10 mM ammonium acetate aqueous solution (v:v, 1:1).

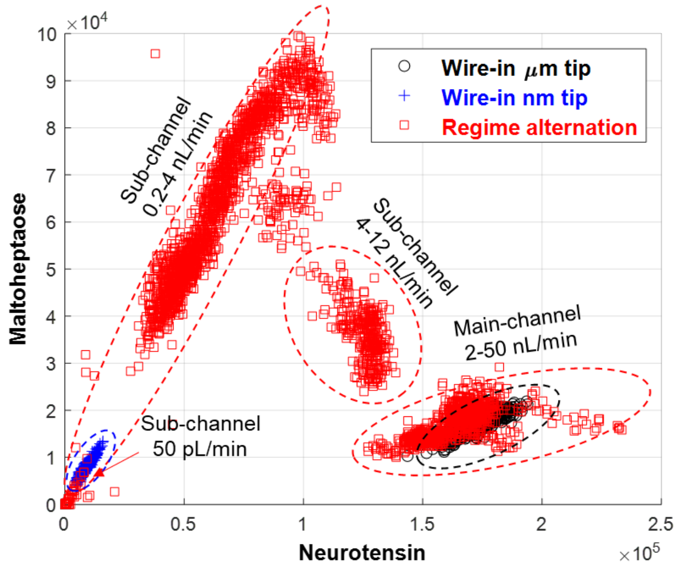

Figure S15. Comparing regime alternation nested-ESI with wire-in nanoESI and picoESI. The integrated intensities of maltoheptaose peaks and neurotensin peaks of each scan were plotted as one data point in the figure. The maltoheptaose to neurotensin ratios may be interpreted by the locations of the points. Data points by wire-in nanoESI using a 4 µm emitter tip clustered in an ellipse along low slope (black). Data points by wire-in picoESI using a 100 nm emitter tip cluster along the slope corresponding to 1:1 ratio (blue). Data points from nested-ESI (red) located in three clusters. The main-channel regime produced data points like that of nanoESI. The sub-channel regime produced two type of data points. Type one data points from lower sub-channel flow rates had M-to-N ratio similar to those of wire-in picoESI but extended to much higher absolute intensities. Type two data points from a higher sub-channel flow rates had M-to-N ratios ranging from 0.2-0.5. Sample being used was 10 µM maltoheptaose and neurotensin in a mixture of methanol and 10 mM ammonium acetate aqueous solution (v:v, 1:1).

12. MS spectra in the main-channel and sub-channel regimes

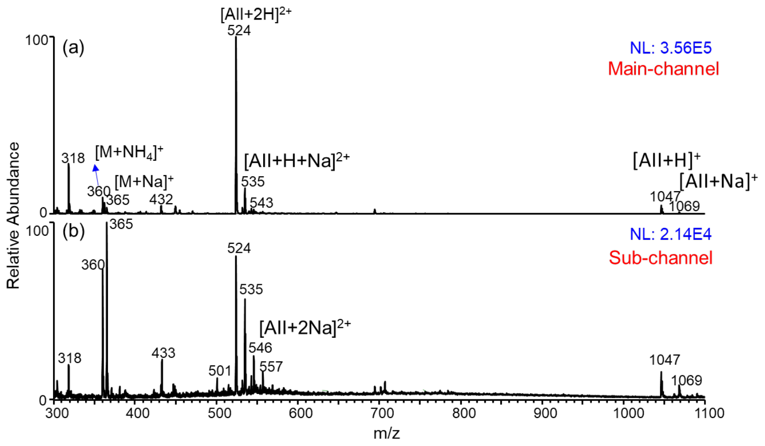

Figure S16. Mass spectra of the mixture of 26 μM angiotensin II (AII) and maltose (M) in (a) main-channel and (b) sub-channel regimes. Experimental conditions: MeOH: water (v:v, 1:1) and 1% formic acid.

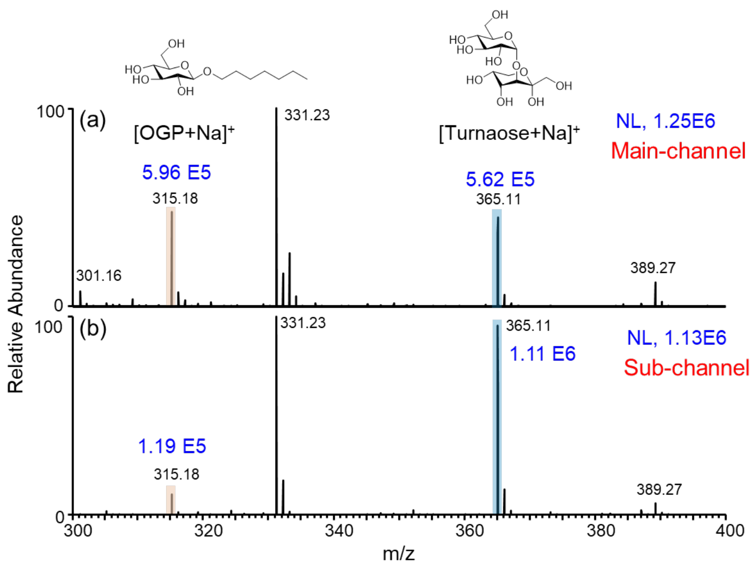

Figure S17. MS spectra for a mixture sample of turanose and n-octyl-glucopyranoside (OGP) in **(a)** the main-channel and **(b)** sub-channel regimes of nested-ESI. A 10 times increase of their relative intensity indicates the difference in surface-to-volume ratio of the initial droplets in the two ESI regimes.[1] Experimental condition: 10 μM turanose and and 1 μM n-octyl-glucopyranoside (OGP) in MeOH and water (v:v, 1:1); MS: LTQ velos Orbitrap.

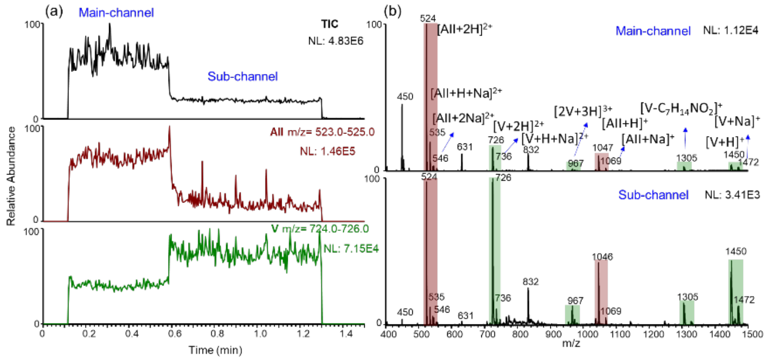

Figure S18. TIC and EICs, MS spectra for a mixture of vancomycin (V) and angiotensin II (AII) in the main-channel regime (12 nL/min) and sub-channel regime (1.1 nL/min) of nested-ESI. The sample solution has 10 µM vancomycin and angiotensin II in 1 mM ammonium acetate aqueous solution.

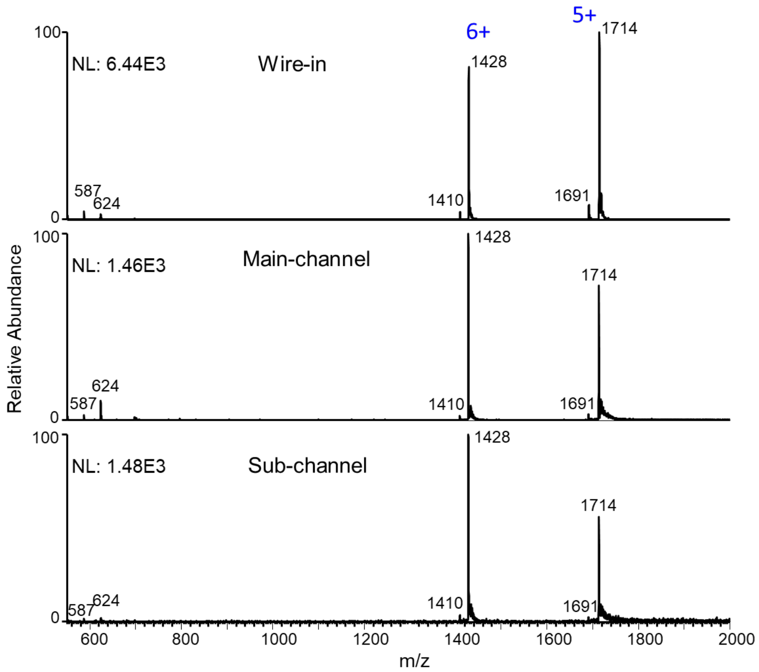

Figure S19. MS spectra of 10 uM ubiquitin in 50 mM aqueous ammonium acetate solution ionized by the positive mode of **(a)** conventional nanoESI (50 nL/min), **(b)** main-channel regime (13 nL/min), and **(c)** sub-channel regime (0.60 nL/min) of nested ESI. In the sub-channel regime, more +6 charge ions were produced for ubiquitin. Both the +5 and +6 charged ions correspond to the folded conformation.[2]

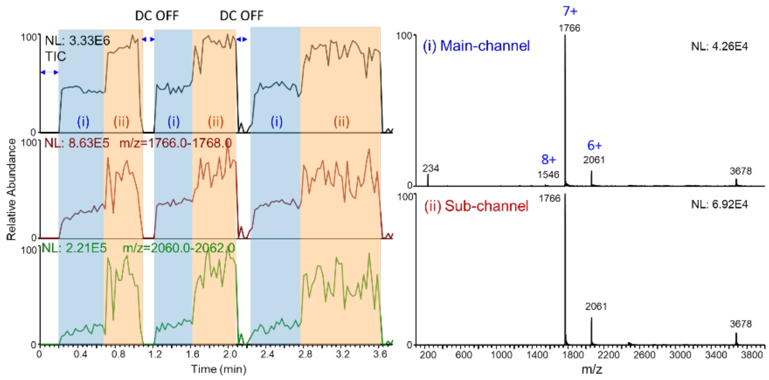

Figure S20. **(a)** TIC, EICs, and **(b)** MS spectra of 50 µM cytochrome c in 50 mM aqueous ammonium acetate solution in main-channel regime (5.2 nL/min) and sub-channel regime (4.0 nL/min) of nested ESI. The ionization efficiency of the protein was significantly higher in the sub-channel regime, despite the minor difference in flow rates.

13. Application I: desalting and decreasing non-specifically adducting by alternating main-channel and sub-channel

Salt adducts is one type of non-specific adducts that complicates data interpretation. The presence of salt also causes ionization suppression. In ESI, a protein in NaCl-containing solution may form adducts ions[3, 4]:

$$[M+(z-n+m)H+nNa+mCl]^{z+}$$

in which **M** represents the neutral analyte, **z** is the charge state, **n** and **m** are integers. Electrospray using smaller emitters tips were found to reduce salt adduct ion signal and non-specific adduct.[5, 6] Here we demonstrate regime alternation as an effective method to reduce and identify non-specific adduct. As shown in the figure below, the TIC shows the different intensities between main-channel regime (i) and sub-channel regime (ii).

In main-channel regime, salt clusters ions (m/z 315-667 with 58 gaps) were generated and cyt c was suppressed. A non-specific adduct with HCl (1772) was produced with significant intensity. The weak HCl adduct was confirmed by the fact it would be lost with minimum energy input during ion isolation by the ion trap. In the sub-channel mode, most of the salt cluster ions disappeared. Cyt c ions became the dominant peak with a 10-times higher absolute intensity. All the produced ions correspond to the folded conformation. The HCl non-specific adduct was not formed.

Despite the complete removal of salt cluster ions for this 5 mM NaCl solution, desalting by sub-channel regime did not work well for more concentrated (100 mM)

salty sample solutions. Calculation suggests that, for a 5 mM solution, a droplet containing less than 2 NaCl molecules need to be smaller than 10 nm in diameter. Removing salt cluster from more concentrated solution would require even smaller initial droplets, which probably exceed the limit of the current setup. This limited desalting capability also confirms that there was no obvious electrophoretic effects[5, 7, 8] during the regime alternation.

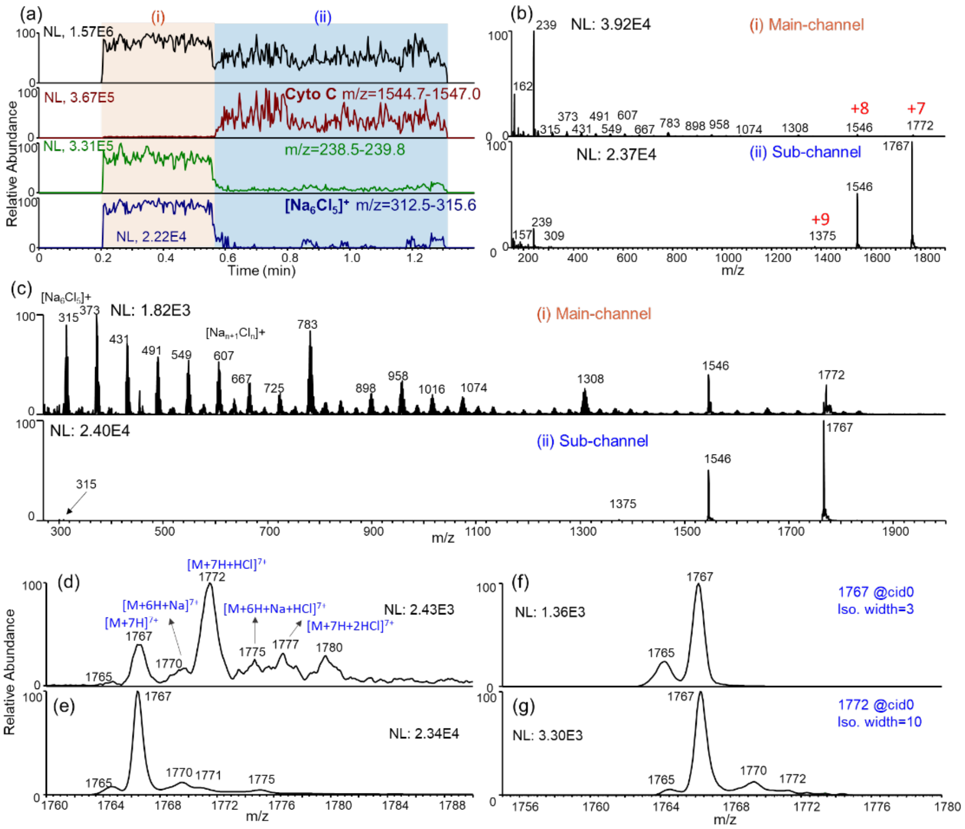

Figure S21. Analyzing 10 µM cytochrome c (MW=12363) in 5 mM NaCl aqueous solution using regime alternation nested-ESI. **(a)** Total ion current, EIC of cytochrome c (m/z=1544-1547), EIC of a solvent background ion (m/z 239), and EIC of salt clusters ($Na_{(n+1)}Cl_n$, m/z 312.5-313.6), in which (i) labels main-channel regime and (ii) labels sub-channel regime. **(b-c)** Full scan spectrum of cyt c in the main-channel and in the sub-channel, zoomed into different mass windows to show the solvent background, salt clusters peaks, and protein peaks. **(d)** Zoom-in of the +7 peak of cyt c indicates significant amount of non-specific adduct was formed in the main-channel regime, while reduced in the **(e)** sub-channel regime. **(f, g)** Isolation of m/z=1766 and m/z 1772 from main-channel regime(d). The minimal energy involved in the isolation of the adducted ion (m/z=1772, isolation window=10.0) could knock off the non-specific adduct in the ion trap.

14. Application II: analyzing and washing online through sub-channel

The operation process was illustrated in Figure S22 below. Firstly, the back end of capillary (length, 40-60 mm; length of tapered tip, 3-5 mm; tip size, 3-5 um) was dipped into solvent (methanol and water, v/v, 1/1) in a 96 well-plate (liquid volume, 200-360 μL; immersed depth, 1-5 mm). In 15 s, sub-channel would be filled to allow solvent accumulation in the emitter tip, which may be emptied by electrospraying before sample loading operation.

Secondly, the emitter tip was dipped (5 mm) under the liquid surface of sample solution to allow sample aspiration into emitter tip. Roughly 5 nL of sample solution entered the tip for a dipping time of 9-12s. During the operations, small amount of solvent (0.5-1.5 nL) accumulated at the tip due to capillary flow. This did not affect the following experiments.

Thirdly, the capillary was mounted in front of MS for nest-ESI. The sample solution was ionized from the main-channel regime, while blank solvent flow from the sub-channel washed the emitter continuously. Analyzing-while-washing was realized for direct ESI analysis. In most cases, the analytes can be cleaned up before the nest-ESI entering the sub-channel model, while regime alternation may be used to further clean the emitter.

More sample solutions may be analyzed by repeating step 1-3. The solvent in the sub-channel typically last for 4-6 analyses, before blank solvent can be added by step 0.

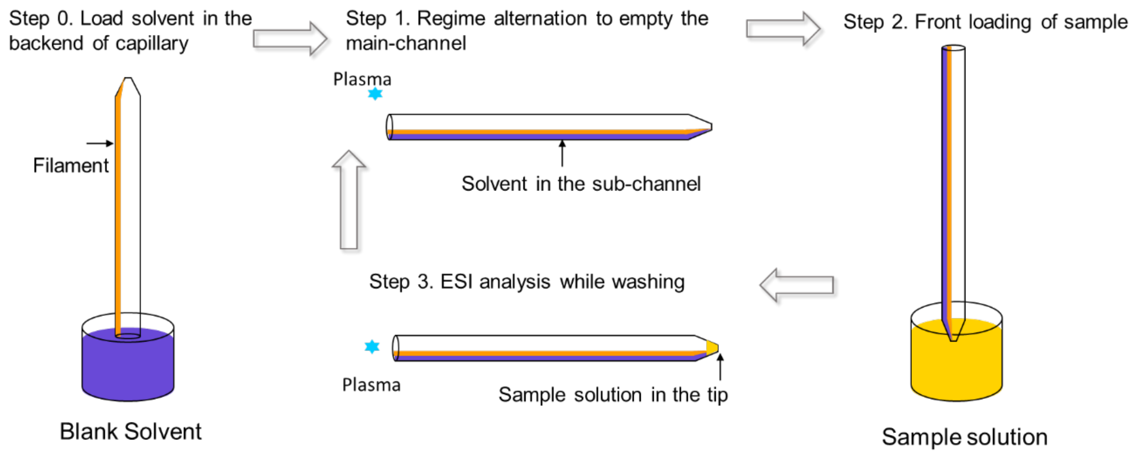

Figure S22. Analysis-while-washing by using the two channels of nested-ESI.

10 μM caffeine aqueous solution (m/z=195) was chosen as another example, as shown in Figure S23. After solvent filled in the subchannel of capillary, the separate operation 1 and 2 were to pause the MS recording to dip the tip of capillary into sample solution about 9-12 s, and then continued detecting. the process of analysis with washing took about less than 0.1 min, the backfilled solvent can last 2.4-3.0 min during each operation. In one operation, the TIC and EICs shows the analyte's signals (m/z=195) were shown firstly, and then gradually decreased because of rinsing; the solvent was continued to flush the tip (as seen the EIC of solvent in the mass range of 233-235). That the analytes cannot be observed in full scan and tandem mass (the relative intensity decreased to at least 10%) is used to prove cleanliness. Mass signals of caffeine and solvent illustrate the analysis is valid and washing is clean without residue.

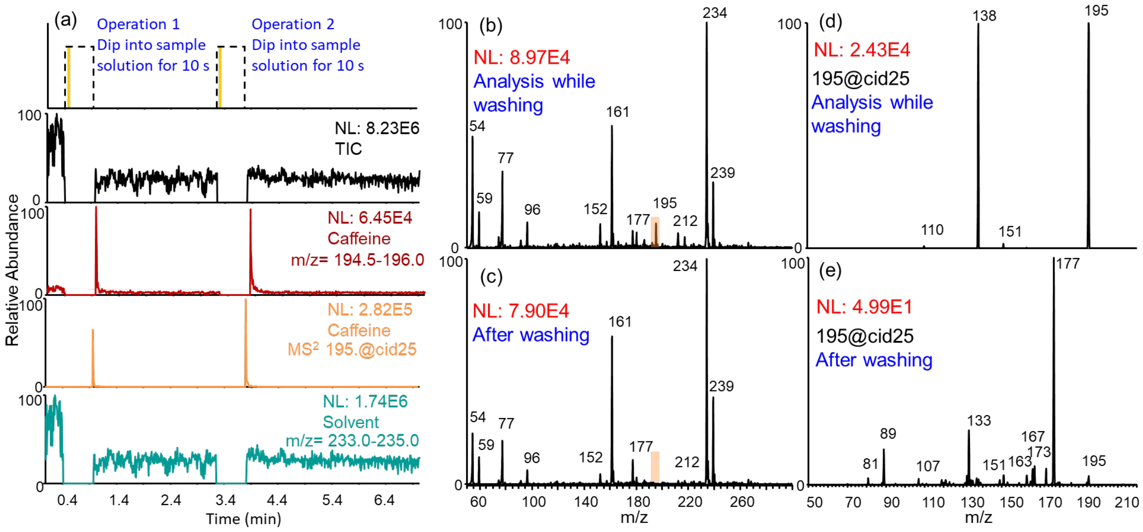

Figure S23. **(a)** TIC, EICs, **(b-e)** mass spectra and tandem mass spectra obtained using analysis while washing by nested-ESI. Sample solution is an aqueous 10 μM caffeine.

As an alternative washing method, aspirating blank solvent from the front end of the emitter tip was tested (Figure S24). 10 μM angiotensin II in aqueous solution was used in this control experiment. As shown in Figure S25, full scans and EIC of angiotensin II in operation 1 indicate the solution at the tip was gradually consumed without any supply from the sub-channel. Then the capillary was removed and dipped into blank solvent for 10 s. The following analysis showed angiotensin II contamination. Even after three such washing operations and spraying for more than 10 minutes,

angiotensin II contamination was still evident. In comparison, the previous washing method which generally takes 0.1-0.5 minutes, was significantly more efficient.

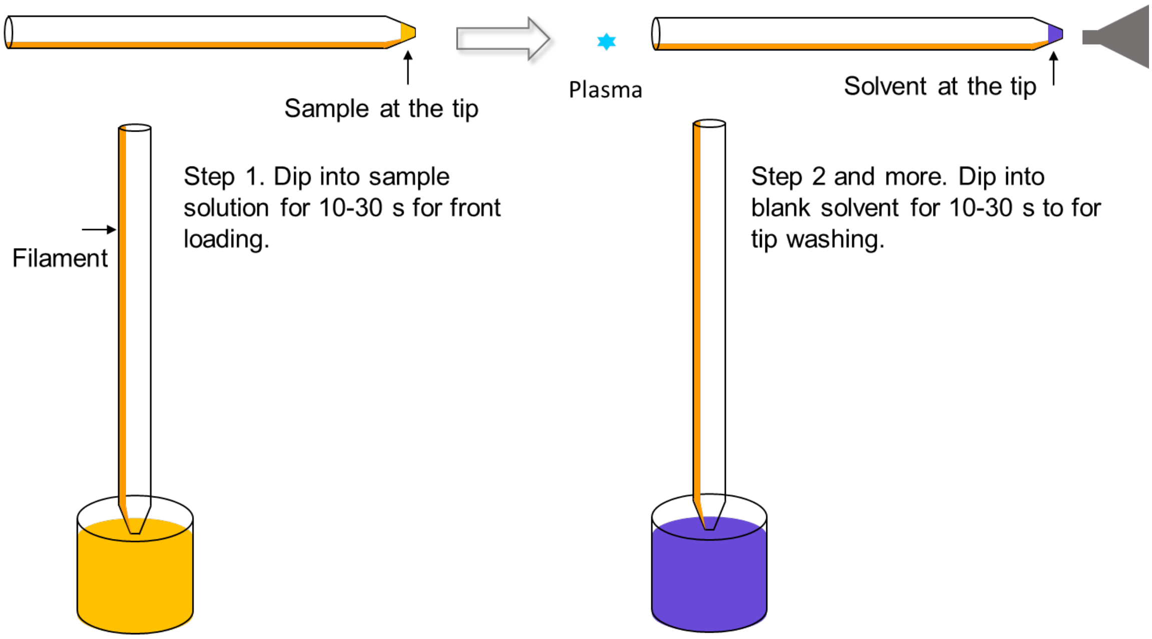

Figure S24. Schematic of an experiment in which blank solvent was added from the front end as an alternative way to wash the emitter tip.

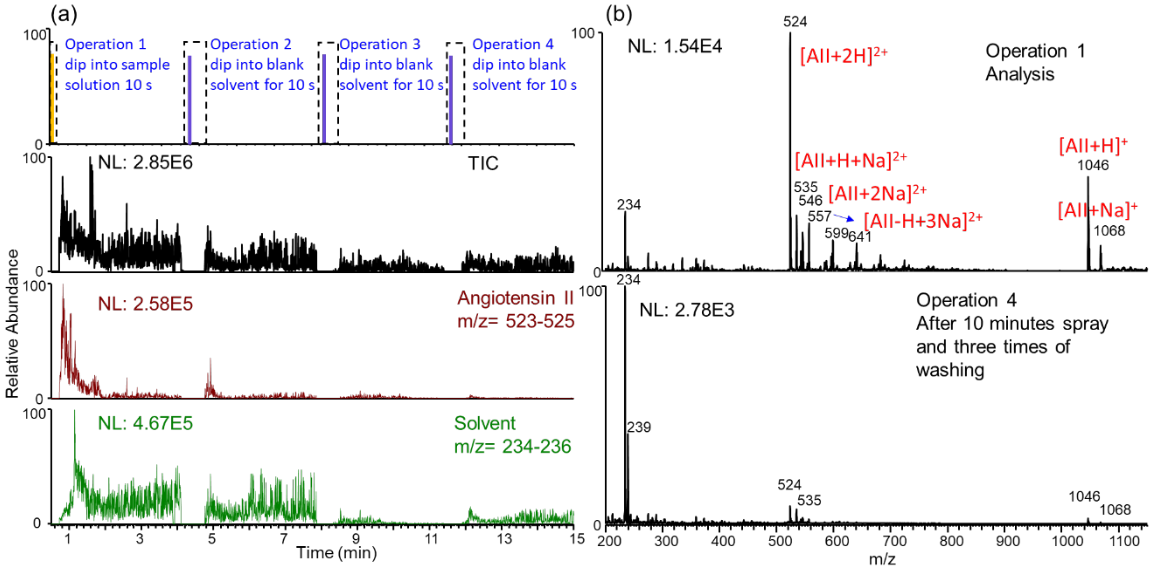

Figure S25. Comparison experiment in which the emitter tip was washed by dipping the emitter tip into bulk solution. The operations were shown in Figure S24. Sample was a 10 µM angiotensin II solution. Carryover contamination was not removed.

Reference

1. A. Schmidt, M. Karas and T. Dulcks, *J. Am. Soc. Mass. Spectrom.*, 2003, **14**, 492-500.
2. L. Konermann and D. J. Douglas, *J. Am. Soc. Mass. Spectrom.*, 1998, **9**, 1248-1254.
3. U. H. Verkerk and P. Kebarle, *J. Am. Soc. Mass Spectrom.*, 2005, **16**, 1325-1341.
4. X. Yue, S. Vahidi and L. Konermann, *J Am Soc Mass Spectrom*, 2014, **25**, 1322-1331.
5. Z. Zhang, C. J. Pulliam, T. Flick and R. G. Cooks, *Anal. Chem.*, 2018, **90**, 3856-3862.
6. Z. Xia and E. R. Williams, *J. Am. Soc. Mass Spectrom.*, 2018, **29**, 194-202.
7. Y. Chen, S. Yuan, Y. Liu and G. Huang, *Anal. Chim. Acta*, 2021, **1141**, 120-126.
8. D. S. Kulyk, D. J. Swiner, T. Sahraeian and A. K. Badu-Tawiah, *Anal. Chem.*, 2019, **91**, 11562-11568.
